# Consumers’ intention to use health recommendation systems to receive personalized nutrition advice

**DOI:** 10.1186/1472-6963-13-126

**Published:** 2013-04-04

**Authors:** Sonja Wendel, Benedict GC Dellaert, Amber Ronteltap, Hans CM van Trijp

**Affiliations:** 1 NIVEL, Netherlands Institute for Health Services Research, P.O. Box 15683500BN, Utrecht, the Netherlands; 2 Erasmus School of Economics, Erasmus University Rotterdam, P.O. Box 17383000, DR Rotterdam, The Netherlands; 3 LEI Consument & Gedrag, Wageningen University, P.O. Box 81306700, EW Wageningen, the Netherlands; 4 Marketing and Consumer Behavior Group, Wageningen University, P.O. Box 81306700, EW Wageningen, the Netherlands

## Abstract

**Background:**

Sophisticated recommendation systems are used more and more in the health sector to assist consumers in healthy decision making. In this study we investigate consumers' evaluation of hypothetical health recommendation systems that provide personalized nutrition advice. We examine consumers' intention to use such a health recommendation system as a function of options related to the underlying system (e.g. the type of company that generates the advice) as well as intermediaries (e.g. general practitioner) that might assist in using the system. We further explore if the effect of both the system and intermediaries on intention to use a health recommendation system are mediated by consumers' perceived effort, privacy risk, usefulness and enjoyment.

**Methods:**

204 respondents from a consumer panel in the Netherlands participated. The data were collected by means of a questionnaire. Each respondent evaluated three hypothetical health recommendation systems on validated multi-scale measures of effort, privacy risk, usefulness, enjoyment and intention to use the system. To test the hypothesized relationships we used regression analyses.

**Results:**

We find evidence that the options related to the underlying system as well as the intermediaries involved influence consumers' intention to use such a health recommendation system and that these effects are mediated by perceptions of effort, privacy risk, usefulness and enjoyment. Also, we find that consumers value usefulness of a system more and enjoyment less when a general practitioner advices them to use a health recommendation system than if they use it out of their own curiosity.

**Conclusions:**

We developed and tested a model of consumers' intention to use a health recommendation system. We found that intermediaries play an important role in how consumers evaluate such a system over and above options of the underlying system that is used to generate the recommendation. Also, health-related information services seem to rely on endorsement by the medical sector. This has considerable implications for the distribution as well as the communication channels of health recommendation systems which may be quite difficult to put into practice outside traditional health service channels.

## Background

Advances in information and communication technology (ICT) allow firms to model complex consumer decision making processes at an increasingly personalized level. These sophisticated systems are used to provide consumers with complex information services such as personalized recommendations to help them find better goods or services in the marketplace [1,2]. Also various health organizations (both public and private) have begun to use sophisticated recommendation systems that offer promising tools to assist consumers in healthy decision making [3-5]. For example Genetic Health (see http://www.genetic-health.co.uk) offers personalized health recommendations to individuals. Specifically, Genetic Health offers advanced commercial applications of personalized gene testing; such as for instance personalized nutrition gene testing (the ‘New Gene Test’) that enables individuals to obtain personalized nutrition advice based on their DNA. Genetic Health then examines polymorphisms (e.g., glucose metabolism) and provides individuals with information that will help them to make lifestyle-related decisions such as for instance how to optimize their diet and regulate their body mass index or how to assist in the body's elimination of serious exposure to toxins. In academia health researchers have also emphasized the importance of personalized health recommendations to support individual consumers in their efforts to successfully adopt healthier eating habits [3], stop smoking [6], or become more physically active [7].

In other sectors, personalized recommendations are also being applied. Consider for instance Amazon.com, which provides consumers with personalized book recommendations on the basis of what other books clients that ordered this particular book also purchased. Also, based on consumer expressions of their present preferences, Amazon.com uses an approach to search and select product descriptions that map well onto the expressed preferences.

In many instances, such as for example in the case of Amazon.com, the recommendation systems are based on relatively neutral and impersonal information provided by the consumer leading to relatively non-intrusive, recommendations. These IT-based recommender systems use relatively simple and non-sensitive consumer input and provide recommendations (output) that are relatively generic, yet very helpful to the consumer. However, in the health sector more in-depth information services are desirable than most current systems provide. Here, recommendation systems also hold considerable potential for personalized advice on the basis of deeper and more complex information disclosed by the customer through so-called “knowledge based recommender applications” [8]. Consider the example of Genetic Health provided above. Based on in-depth knowledge of an individual’s genetic information, increasingly personalized dietary advice can be provided through information systems that incorporate this state-of-the-art scientific knowledge [9]. Yet, this type of recommendation typically requires the exchange of privacy sensitive information between individuals and the firm, a process that is not easily supported by information technology alone [10]. Rather such recommendation systems can greatly benefit from close collaboration with intermediaries [11], such as general practitioners or other (health) professionals that facilitate the information exchange [12]. In particular, intermediaries can facilitate extensive interactions between consumers and firms (to communicate consumer needs and deliver personalized recommendations) and help increase consumer trust and involvement in the service process [13,14]. In the field of personalized genomics the important role that intermediaries play has been illustrated. In particular a study by Gollust et al. [15] shows that more than 90% of early adopters of personalized genomics are willing to share the results of a personalized genome test with their general practitioner.

To date, most research pertaining to personalized recommendations has focused on developing new methods to improve the quality of the underlying characteristics of the system (hereafter named information system) such as for instance the type of recommendation provided or has analyzed how consumers make product choices when provided with personalized recommendations [16,17]. We extend this research by providing a broader perspective of a recommendation system that is based on more privacy sensitive information and also reliant on consumer interactions with intermediaries. Therefore, we first investigate whether consumers’ intention to use a health recommendation system to get personalized nutrition advice is influenced by the information system itself as well as by the intermediaries that facilitate the interaction (for instance a general practitioner). Second, we investigate the cost-benefit trade-offs that individuals make when deciding whether or not to use such a recommendation system and conceptualize this as a process in which individuals evaluate the personal information and effort that they contribute in exchange for a useful and enjoyable personalized recommendation by the firm [18,19]. More specifically, we propose that individuals are affected not only by the characteristics of the information system used to provide the personalized recommendation, but also by the intermediaries that facilitate the information exchange both at the level of inputting information and on extracting the personalized advice. In addition to facilitating the interaction with clients at the input and output level of the information system, advice by intermediaries to use a health recommendation system that provides personalized nutrition advice may also essentially change the individual’s intention to use the recommendation system itself. Therefore, as a third step, we also investigate the role and impact of intermediaries on individuals’ intention to use recommendations systems. Individuals typically rely strongly on their general practitioners to guide their health-related decisions [20]. In particular, an advice of relevant intermediaries (such as a general practitioner) may change the individual’s mindset in the evaluation of a health recommendation system from a more enjoyment oriented hedonic focus to a more utilitarian focus on instrumentality [21]. Thus, intermediary endorsement may moderate consumer decision making and evaluation process of health recommendation systems. Therefore we also explore how the role of intermediaries affects consumers’ intention to use a health recommendation system compared to when they use it out of their own interest [22].

### Theory and hypotheses

#### Recommendation systems

Recommendation processes have mainly been conceptualized emphasizing the operational process and the firm’s role as a producer of recommendations [23,24]. Essentially, such recommendation processes are based on the efficient intake of information from the consumer, matching of that information to advanced knowledge bases for the translation into a personalized advice, and mutual learning on the basis of effectiveness of the translation process in terms of customer and firm satisfaction with the exchange process [23,25]. We argue that such restricted conceptualization may be appropriate for relatively simple “feature search and match” recommendation systems, where a relatively low stake exchange of information can be assumed, but that for more advanced knowledge based recommendation systems such smooth flux in information exchange cannot be taken for granted. Knowledge based recommendation systems differ in a number of ways from the simpler “feature search and match” systems [26]. First, the information technology behind them is substantially more complex and analytical due to contingencies in the input–output matching process. Second, at the input level advanced knowledge based systems typically require the exchange of detailed, fuzzy, and sensitive information between consumers and firms - as in personal health advice tailored to an individual’s genetic disposition or a personalized financial advice that matches an individual’s retirement plans. Finally, at the recommendation (output) level generic solutions may be inadequate for these systems as consumers expect a much more personalized and non-casual advice, both in terms of content and mode of delivery. All in all, knowledge based recommendation systems are “high stake” for consumers and likely to be complex and impactful, rather than transparent and casual as in simpler feature-search-and-matching recommendation systems.

The perceived uncertainty in the customer–firm relationship, inherent in knowledge based recommendation systems, provides a potential barrier to the adoption of such recommendation systems [26,27]. Rather than presuming the customer as a relatively passive provider and receiver of information, knowledge based recommendation systems require a more integrative approach that explicitly includes customer relationship management to build the trust required to lock-in the customer as a co-designer of the personalized recommendation.

**Figure 1:**
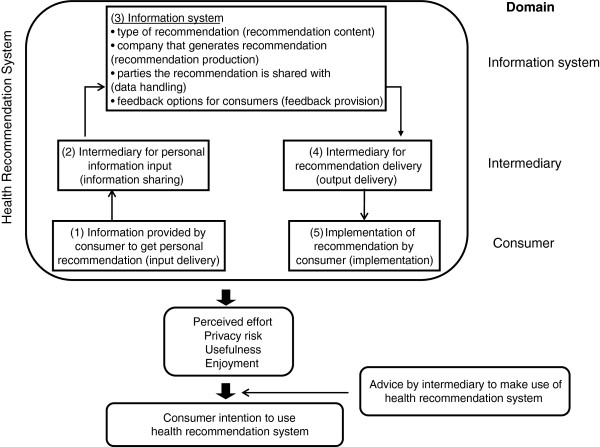
Conceptual model.

Such recommendation systems (see Figure 1) differentiate from pure information systems in that they explicitly include the customer relationships management through the intermediary’s role in the recommendation process. We classify the different aspects of a health recommendation system in three main domains (see Figure 1). First, the *information system domain* refers to the information system and the various processes undertaken by the firm(s) to generate a recommendation on the basis of the consumer’s personal information. Second, the *intermediary domain* comprises the processes by which consumer and firm interact. Third, to control for the fact that different consumer actions may be necessary, we also add a *consumer activity domain* in the conceptual model. This domain captures what type of information the consumer needs to provide as input for the personalized recommendation, and the actions that the consumer needs to undertake to implement a recommendation in his or her daily life.

Intermediaries such as general practitioners play a crucial role in the information flux between consumers and the firm by facilitating (a) the release of personal information on the part of the consumer, (b) the delivery of tailored nutritional advice to the consumer, and (c) trust in procedural justice in the use (and protection) of the privacy sensitive information. Combining the use of information systems with personal interactions between customers and intermediaries is often highly beneficial [11]. This leads to the following hypothesis on consumers´ intention to use a health recommendation system:

H1: Consumers’ intention to use a health recommendation system depends both on the information system and on the intermediaries that are active.

#### Consumer intention to use a health recommendation system as a cost-benefit trade off

The customer’s decision to use a health recommendation system can be adequately modeled as a trade-off between perceived costs and benefits [28]. To describe the cost-benefit trade-offs we draw on psychological contract theory which has been developed in the organization literature to describe people’s beliefs in the reciprocal obligations between employees and organizations [18,29]. In the context of health recommendation systems a similar structure of expectations between the consumer and the firm exists such that the consumer perceives the input he or she provides obligates the firm to return a higher quality, tailored recommendation. Thus, we conceptualize the underlying cost–benefit trade-offs that consumers make in evaluating health recommendation systems as a type of psychological contract, in which consumers contribute personal information and effort in exchange for a more useful, tailored recommendation by the firm. Benefits refer to the instrumentality of the engagement (i.e. usefulness in terms of contribution to personal health and wellbeing) as well as intrinsic enjoyment of doing so, and costs involve the perceived effort as well as the uncertainty and risk perception with releasing the sensitive information, also known as the privacy calculus [30].

First, the degree to which a person believes that using a health recommendation system takes a lot of effort (*effort*) constitutes an important first cost aspect that consumers likely consider [31]. Consumers benefit from being able to interact in person with service staff. These interactions reduce the difficulty for consumers in using the recommendation process by offering meaningful feedback and allowing for direct responses that can be used to immediately clarify potential difficulties [13].

Second, providing in-depth personal information also involves the potential risk of misuse of the information by the firm. Therefore, the next cost component we propose is that consumers take into account is the degree to which consumers believe that the health recommendation system is risky when providing sensitive information (i.e., *privacy risk*). Consumers are very concerned about their privacy, especially when it comes to health-related services, the context of our empirical study, and generally are reluctant to provide personal information [32,33]. Recent trends in information technology that enable companies to collect more accurate and detailed personal information likely have further increased consumers’ privacy concerns [34]. Personal interactions with intermediaries however can overcome these concerns and instill greater confidence with consumers that privacy risks are low [35].

Third, in terms of benefits the *usefulness* of a new technology, or the degree to which a person believes that using a health recommendation system is beneficial in achieving his or her desired outcomes, is an important benefit identified in consumer evaluations of new technologies [36,37]. With the proliferation of technology and the Internet, this benefit also appears with regard to the end-user information technologies that are relevant for the context of personalized recommendations [38,39]. Personal interactions with intermediaries however further allow consumers to better understand product and service characteristics and how they relate to their particular needs, thus increasing the usefulness of the recommendation process [11,40].

Fourth, an additional benefit that may compensate the anticipated effort by the consumer is the anticipated *enjoyment* of using the health recommendation system. In research on technology-based self-service, Dabholkar and Bagozzi [41] demonstrate that enjoyment significantly influences consumers’ attitude toward a technology-based self-service. Findings by Van der Heijden [42] provide further support for the impact of perceived enjoyment on consumers’ attitude in the context of Web site evaluations. In-person interactions such as those with intermediaries also increase the enjoyment of the interaction process [43]. Therefore, we expect that enjoyment, which we define as the degree to which a person believes that using a health recommendation system will be an enjoyable experience, also drives consumers’ intention to use such a system. Jointly, these considerations lead us to formulate the following hypotheses about the effects of intermediaries on consumers’ intention to use a health recommendation system:

H2: The effect of intermediaries and information systems on consumers’ intentions to use a health recommendation system is mediated by consumers’ perceptions of (a) the effort, (b) privacy risk, (c) usefulness, and (d) enjoyment involved in using the health recommendation system.

#### Intermediary participation advice and consumer cost–benefit focus

Though in a general sense consumers’ cost-benefit perceptions determine their intention to use a health recommendation system [37], previous research also shows that consumers’ cost-benefit focus may be context dependent [44-46]. We anticipate that consumers’ intention to use a health recommendation system specifically depends on the setting in which they are introduced to this system. In particular, we propose that when a health recommendation system is introduced in a more formal setting in which consumers are given the advice by the intermediary to use the system, their cost-benefit trade-offs differ compared to a setting in which such formal endorsement of the intermediary is absent and consumer usage of the system is self-directed (e.g., through curiosity). More specifically, when a health recommendation system appears in a setting in which consumers feel obliged to make use of it, the system mainly represents a means to an end. We expect that this causes consumers to feel a stronger extrinsic motivation to use it [47] compared to when such external pressure is absent. In contrast, when introduced to the system in a more spontaneous setting, we expect that they are more likely to view using the health recommendation system as a consumption experience in its own right (e.g., to explore new, healthier food options) [48].

The effect of extrinsic versus intrinsic motivation influences people’s purchase [49] and technology adoption decisions [50-53], and a recent literature review by Novak et al. [22] illustrates the coherence of this distinction through several related types of consumer behavior. One of the main behavioral differences between the two motivation categories stems from the stronger goal orientation and lower emphasis on experiential evaluations that is associated with extrinsically motivated behaviors compared to intrinsic motivation [54]. Thus, utilitarian and goals correlate with extrinsically motivated behavior and hedonic goals with intrinsically motivated behavior [21].

We expect that a similar shift from a utilitarian to an experiential focus is relevant in health recommendation system evaluations. Due to the differences in motivation when given advice to participate in such a system versus when participating out of one’s own interest, we expect that consumers cost-benefit trade-offs are also different. In particular we expect that consumers who are advised to use a health recommendation system by an intermediary find the system’s usefulness more important and its enjoyment less important compared to consumers who use a health recommendation system out of their own interest. This difference is particularly relevant in the domain of health, where consumers typically are either advised to use an information system (e.g., by a doctor with the aim to achieve better health) in which case the usefulness of the health recommendation system becomes more important, or may use the information system out of their own interest (e.g., after having heard from a friend about recommendation systems and getting curious) and the enjoyment of the system becomes more important [20,55-57]. A number of studies in the area of genetics also support this distinction we make and show that consumers’ curiosity is a main motivator for making use of personalized genome testing [24,50,58]. Therefore, we hypothesize that:

H3: When an intermediary supports the health recommendation system in an advice to the consumer this (a) *increases* the impact of perceived usefulness and (b) *decreases* the impact of perceived enjoyment on their intention to use the health recommendation system, compared to when consumers use the health recommendation system out of their own interest.

## Methods

We test our hypotheses in the area of personalized nutrition recommendations since they represent a key example in the health sector of a type of personalized recommendation system that provides a particularly promising tool to assist consumers in their decision making [3]. Data collection for our study involved 204 respondents from a large, representative national consumer panel in the Netherlands who responded to hypothetical scenarios about different personalized nutrition recommendation systems. The study is approved by the institutional ethical committee at Wageningen University.

We test our hypotheses in the context of these types of applications and ask consumers to evaluate hypothetical scenarios of how they might obtain personalized health nutrition recommendations to improve their health by changing their food intake and meal preparation.

### Scenario development

In the first step in our research, we developed an in-depth, qualitative understanding of experts’ from academia and business and consumers’ views about how to provide consumers with specific behavior recommendations and tailored food and nutrition intake advice. Based on these discussions we operationalized health recommendation systems in terms of a total of eight steps across the information system, the intermediary domain, domain, and the consumer activity domain. Each step is defined in terms of one of three realistic options (see Table 1 and Additional file 1).

**Table 1:** Options for the different health recommendation system domains

**Options**	**Domain**	**Options**	**Domain**
Recommendation content	*Information system*	Input delivery	*Intermediary*
• At ingredient level		• Through fitness club	
• At food product group level		• Trough general practitioner	
• Special branded products		• Through hospital	
Recommendation production	*Information system*	Output delivery	*Intermediary*
• By commercial food company		• Through email	
• By insurance company		• Through fitness club	
By government nutrition center		• Through general practitioner	
Data handling	*Information system*	Information sharing	*Consumer activity*
• Fully anonymous		• Blood composition	
• Shared between patient and GP		• Dna/genetic makeup	
• Available to commercial food companies		• Food consumption habits	
Feedback provision	*Information system*	Implementation	*Consumer activity*
• No feedback for verification		• Incorporated in usual meals	
• Option of feedback for verification		• Specific products added to regular meal	
• Obligatory feedback for verification		• Requiring preparation of individually adjusted meals	

We pretested the options in a qualitative study with 11 consumers with whom we conducted individual open interviews during which we discussed the realism and relevance of the options, as well as their interpretations and comprehension. Participants indicated one key potential interaction between the type of personalized information available and the integrity of the data handling by the firm. Therefore we allowed for this interaction in the hypothetical scenarios constructed for the main survey. However, in the estimations of our model the interaction effect was not significant and therefore we exclude it from the reported results. On the basis of these interviews, we refined and finalized the proposed options for each step, as we present in Table 1.

The interviews also resulted in the formulation of the two intermediary advice contexts for the main survey. The required participation condition reads as follows: “You went to your general practitioner for your regular check-up, and your general practitioner advised you that you would feel better if you used an information service that provides personalized recommendations about healthy eating and cooking.” The condition based on participation out of the consumer’s own interest states, “Someone you know has mentioned to you that it is possible to obtain personalized recommendations about healthy eating and cooking, and you would like to try this service.”

### Scale items

The objective of this pretest was to validate the scale items from the literature for use in the empirical context of our research. We randomly assigned - 108 graduate and undergraduate students who received a small monetary compensation for participating - to three different options for intermediaries, information systems, and consumer activity control variables, and they evaluated the options in terms of their perceived costs and benefits and their intention to use the health recommendation system for each option. The evaluation measures all use nine-point semantic differential scales.

The scale items drawn from the literature performed well to very well in the empirical context of our study, with Cronbach’s alphas ranging from 0.87 to 0.94. The exceptions, two items from the usefulness scale, do not appear in the final analysis because of their low item-to-total correlations. On the basis of this approach, we determined the scale items for use in the main survey. We measured usefulness with three items [59] that asked whether the health recommendation system (1) was not useful/useful, (2) was not useful/useful to improve nutrition, and (3) would not/would influence purchases. For effort, we also employed three measures [39] that indicated whether the personalized health recommendation system (1) was difficult/easy to understand, (2) was difficult/easy to learn how to use, and (3) made it difficult/easy to remember what to do. Similarly, the three enjoyment measurement items [41] indicated whether the health recommendation system was (1) not interesting/interesting, (2) not entertaining/entertaining, and (3) not enjoyable/enjoyable. To address the health recommendation system’s privacy risk, we asked respondents to respond to the following three items [60]: (1) I feel insecure/secure about giving up personal information, (2) I feel insecure/secure about giving up information about my health, and (3) the health recommendation system is not safe/safe. Finally, for intention to use, we used two semantic differential scales in which respondents could indicate whether it were likely/unlikely or possible/impossible that they would use the particular health recommendation system [41].

### Main study: sample and procedure

After we had developed the scenarios and completed the pretest, we continued to the data collection step, which was managed by a professional market research agency that recruited 204 respondents from a large, representative national consumer panel in the Netherlands. Respondents were invited to the central test facility for a computer-based task, which constituted part of a larger survey that took an average of one hour to complete. The sample distribution included 50.5% women and 46.1% men (3.4% missing), with an average age of 38.3 years, and ranging from 18 to 64 years. These distributions are comparable to the gender and age distribution in the Dutch population. Of the respondents, 44.9% had completed a higher education degree (bachelor’s degree or higher). Furthermore, 22.4% lived alone, 31.1% lived in a household of two, and 46.4% lived in a household of three or more people. As compared to the Dutch population, our sample on average consists of higher educated respondents, fewer people living alone, and more people living in a larger household (three or more people). In the computer-based task, respondents evaluated three randomly selected scenarios from a full factorial design describing all options for all steps (3^8^ full factorial). The average occurrence of an option was 204 times, with a maximum occurrence of 220 and a minimum of 177 times. This distribution illustrates that the various options appear approximately equally across the scenarios. The scenarios depict hypothetical health recommendation systems (see Additional file 1) each of which offers a full profile description of the eight steps identified in Figure 1 and defined by one of the three options (see Table 1). The instructions provided the respondents with introductions to one of two (hypothetical) usage advice contexts for the health recommendation system. We also include three scale items in the survey to measure the perceived realism of the task. The items all achieve average ratings of greater than three on a five-point Likert-type scale (disagree to agree), which indicates that respondents considered the task realistic. Respondents’ rated all cost-benefit items and their intention to use the health recommendation system for each scenario. Thus, we obtain a total of 612 (3 × 204 respondents) complete scenario evaluations, split equally across the two contexts (required participation versus participation out of one’s own interest).

### Analysis approach

First, we estimate a regression model with consumers’ intentions to use a health recommendation system as dependent variable and the options in the intermediary, information system, and consumer activity domains as independent variables. We test H1 by determining if the parameter estimates for the different options in the intermediary and information system domains are significant. If so, we find support for our claim that these two domains both drive consumers’ intentions to use health recommendation systems.

Second, to test H2 we run a series of additional regression analyses. In particular, we investigate if the cost-benefits of perceived effort, privacy risk, usefulness, and enjoyment are also driven by variations in the intermediary and information system domains, and if these cost-benefits in turn mediate the effect of the domain options on consumers’ intention to use a health recommendation system. Thus, we first estimate four regression models with as independent variables the specific options used for each domain, whereas the dependent variables are the consumers’ evaluations of each cost and benefit. Next, we estimate a regression model in which consumers’ intention to use the health recommendation system is modeled as a function of their cost-benefit evaluations. Finally, we estimate a model where consumers’ intentions to use the health recommendation system are a joint function of the options in each domain as well as the consumers’ cost-benefit evaluations. Following Baron and Kenny’s [61] mediation analysis approach we conclude that (partial) mediation occurs when: (a) the effects of the health recommendation system options on consumers’ intention to use a health recommendation system are significant, (b) the effects of the options on consumers’ evaluations of the cost-benefits are also significant, and (c) the effects of the options on consumers’ intentions to use a health recommendation system are significantly lowered if the cost-benefits evaluations are also taken into account in the same regression model.

Third, we test H3, the hypothesized interaction effect of intermediary advice context (advice to participate vs. participation out of the consumers’ own interest) with consumers’ cost-benefit evaluations for usefulness and enjoyment on consumers’ intention to use the health recommendation system. To do so, we include this interaction effect in the regression model of consumers’ intention to use a health recommendation system with the consumers’ cost-benefit evaluations as dependent variables. If the interaction effects are significant and in the expected direction, we find support for our hypotheses.

In all our estimations we apply a fixed-effects specification for each regression model to allow for unexplained heterogeneity between respondents and the repeated measures nature of the data (i.e., three scenario evaluations observed for each person). The fixed effects model also allows us to correct for the fact that the different respondents saw different random sets of scenarios which may generate different average cost-benefit scores per respondent.

## Results

### Scale performance

We first examine whether the items used to measure the four consumer cost-benefits and consumer intention to use the health recommendation system might measure the same underlying construct or if they are –as expected- related but distinct factors. The fit of the one-factor model is very bad (χ^2^ (77) = 3264.37, *p* < 0.001; comparative fit index [CFI] = 0.70, nonnormed fit index [NNFI] = 0.64, root mean square error of approximation [RMSEA] = 0.26, and adjusted goodness-of-fit index [AGFI] = 0.40), forcing us to reject the simplified one-factor model. Next, we estimate the hypothesized five-factor model, which provides a good fit (χ^2^ (67) = 204.93, *p* < 0.001; CFI =0 .98, NNFI = 0.98, RMSEA = 0.059, and AGFI = 0.93). Moreover, the fit of the five-factor model is dramatically and significantly (Δχ^2^ = 3059.44, *p* < 0.001) better than that of the one-factor model. All factor loadings are significant (t-values greater than 13.86), and all completely standardized loadings are greater than 0.56, with an average of 0.84. These findings support the convergent validity of the measures. Cronbach’s alphas are 0.80, 0.88, 0.88, 0.90, and 0.93 for effort, privacy risk, usefulness, enjoyment, and intention to use the health recommendation system, respectively. We follow the approach by Fornell and Larcker [62] to test the discriminant validity of our measures and test if the average variance extracted (AVE) of a latent construct must be greater than the squared correlations with other latent constructs. The estimates of the AVE are 0.82, 0.71, 0.82, 0.85, and 0.93 for effort, privacy risk, usefulness, enjoyment, and intention to use the health recommendation system, respectively, and exceed the squared correlation of these constructs. We therefore conclude that discriminant validity is good and obtain a composite score for each separate construct by averaging the appropriate scale items.

### Hypotheses testing

The results for H1 appear in the first column of Table 2 (“Options model”), which shows the effects of the different options of the health recommendation system on consumers’ intention to use the system. Options from both the intermediary and information systems domains significantly influence consumers’ intention to use a health recommendation system providing support for H1. The results also show that changes in the options in the consumer activity domain (i.e., the control options under information sharing and implementation) influence the cost-benefits of effort, privacy risk, usefulness, and enjoyment less than do changes in the options in the intermediary and information system domains.

**Table 2:** **Effects of options, cost-benefit perceptions, and participation advice on intention to use a health recommendation system**^**a**^

**Cost-benefits**		**Options model**	**Cost-benefits model**	**Mediation test model**
Effort			0.00	0.00
Privacy risk			−0.20**	−0.20**
Usefulness			0.30**	0.30**
Enjoyment			0.46**	0.46**
Impact of participation advice on the effect of usefulness			0.13**	0.13**
Impact of participation advice on the effect of enjoyment			−0.10*	−0.09
*Recommendation system options*	*Domain*			
*Recommendation content: Base = Ingredients*	Information system			
Product groups		0.07		−0.04
Product brands		−0.11		−0.05
*Recommendation production: Base = Commercial food company*	Information system			
Insurance company		−0.20**		−0.04
Governmental nutritional center		−0.03		−0.03
*Data handling: Base = Fully anonymous*	Information system			
Shared with general practitioner		−0.01		−0.04
Available to commercial food company		−0.16**		−0.07*
*Evaluation: Base = No feedback*	Information system			
Optional feedback		0.14*		−0.02
Obligatory feedback		0.13*		0.02
*Communication: Base = Fitness club*	Intermediary			
Through general practitioner		0.16**		−0.05
Through hospital		0.09		−0.04
*Delivery: Base = Through e-mail*	Intermediary			
Through general practitioner		−0.07		−0.02
Through fitness club		−0.19**		−0.03
*Information sharing: Base = Blood composition*	Consumer activity			
DNA/genetic makeup		−0.10		−0.04
Food consumption habits		0.05		−0.03
*Implementation: Base = Usual meal*	Consumer activity			
Addition to regular meal		0.01		0.04
Separate cooking		0.00		0.04
Model Fit		R^2^ = 0.38	R^2^ = 0.81	R^2^ = 0.81

^**a**^ Estimates of regression model with individual-specific fixed effects.

*Significant at *p* < 0.05;.**Significant at *p* < 0.01.

The main effects of consumers’ cost-benefit perceptions also appear in Table 2 (second column “Cost-Benefits model”) and indicate that consumer perceptions of cost-benefits are strong predictors of intention to use a health recommendation system (R^2^ = 0.81). Although greater effort does not decrease consumers’ intention to use the health recommendation system, greater privacy risk has a significant effect as expected (*p* < 0.001). Also, greater usefulness is a strong and significant predictor of consumers’ intention to use the system (*p* < 0.001). Furthermore, consumers’ intention to use the health recommendation system significantly increases with greater enjoyment (*p* < 0.001).

To test for the hypothesized mediation of the recommendation system option effects on consumers’ intention by cost-benefit evaluations (H2), we estimate the following additional models. First, we investigate if the system options exert significant effects on the mediators (cost-benefit perceptions). The outcome of these models are reported in Table 3, and we find many significant effects of health recommendation system options in the intermediary and information systems domains for each of the four cost-benefit perceptions. Second, we investigate if the effect of the recommendation system options on consumers’ intention to use the health recommendation system reduces when we incorporate the mediating variables as covariates in the model [61]. Column 3 in Table 2 shows the results of the regression model that includes both the cost-benefit and recommendation system options (“Mediation test model”). We find that all except one effect of the recommendation system options become insignificant when cost-benefits are introduced as covariates; the exception occurs when in the data handling stage, personal information is made available to a commercial food company. Thus, we find evidence for mediation for all recommendation system option effects that are significant in the options only model, and more specifically that all but one of these effects are fully mediated. These findings clearly support H2.

**Table 3:** **Effects of health recommendation system options on cost-benefit perceptions**^**a**^

	**Domain**	**Effort**	**Privacy risk**	**Usefulness**	**Enjoyment**
*Recommendation content: Base = Ingredients*	Information system				
Product groups		−0.09*	−0.08	0.06	0.14*
Product brands		−0.01	−0.02	−0.11	−0.08
*Recommendation production: Base = Commercial food company*	Information system				
Insurance company		0.07	0.13*	−0.17**	−0.18**
Governmental nutritional center		−0.02	−0.08	−0.05	−0.04
*Data handling: Base = Fully anonymous*	Information system				
Shared with patient and general practitioner		−0.04	−0.07	−0.01	0.04
Available to commercial food company		0.06	0.14*	−0.08	−0.10
*Evaluation: Base = No feedback*	Information system				
Optional feedback		−0.15**	−0.18**	0.19**	0.17**
Obligatory feedback		−0.12*	−0.13*	0.09	0.13*
*Communication: Base = Fitness club*	Intermediary				
Through general practitioner		−0.15**	−0.23**	0.24**	0.21**
Through hospital		−0.10*	−0.18**	0.17**	0.11
*Delivery: Base = Through e-mail*	Intermediary				
Through general practitioner		0.03	0.06	0.01	−0.10
Through fitness club		0.10*	0.13*	−0.17**	−0.18**
*Information sharing: Base = Blood composition*	Consumer activity				
DNA/genetic makeup		0.06	0.11*	−0.04	−0.06
Food consumption habits		−0.04	−0.13*	0.06	0.08
*Implementation: Base = Usual meal*	Consumer activity				
Addition to regular meal		0.00	0.01	−0.04	−0.02
Separate cooking		0.08	−0.04	−0.06	−0.05
Model Fit		R^2^ = 0.58	R^2^ = 0.43	R^2^ = 0.33	R^2^ = 0.30

^**a**^ Estimates of regression model with individual-specific fixed effects.

*Significant at *p* < 0.05.

**Significant at *p* < 0.01.

H3a and H3b posit moderating effects of the intermediary advise context (advice to participate versus participation out of one’s own interest, coded as 1 versus 0) on the impact of the benefits usefulness and enjoyment respectively on consumers’ intention to use a health recommendation system. To test these hypotheses we allow for an interaction of the intermediary advice context with the benefits usefulness and enjoyment, along with the main effects of the recommendation systems’ cost-benefits. The results are also reported in the cost-benefits model in Table 2 (second column, “Costs-Benefits model”). The results clearly support H3a and H3b. We find that the effect of usefulness on consumers’ intention to use the health recommendation system is greater when the intermediary advices participation in the recommendation system (p <0.01) and that the effect of enjoyment is smaller in the advised participation context (p<0.05).

### Further analysis

We conducted a further analysis to investigate if the insignificant effect of effort on consumers’ intention to use a health recommendation system might be explained by a second mediation effect, such that the impact of effort on consumers’ intentions is mediated by perceived usefulness. Previous research suggests at least partial mediation of the effect of effort or its counterpart ease of use [31,63]. Therefore, we conduct a second Baron and Kenny [61] mediation test to explore if the effect of effort on consumer intention to use a health recommendation system is mediated by the perceived usefulness of the system. We eliminate usefulness in the intention to use the health recommendation system model and find that effort becomes significant (*p* < 0.001). In addition, we estimate a model relating effort to usefulness and find a significant positive effect (*p* < 0.001). Therefore, a mediation effect of effort on consumers’ intention to use a health recommendation system exists through usefulness, which explains our finding of no effect for effort on intention to use a health recommendation system.

## Discussion

Our findings provide support for the proposed conceptual model and hypotheses, and managerial guidance to firms and public policymakers that wish to promote the use of complex knowledge based recommendation systems by consumers. Organizations that wish to promote knowledge based recommendation systems to potential customers should consider multiple steps for communicating and introducing these systems in the broader perspective of recommendation systems to consumers. In particular, intermediaries play a crucial role in how consumers evaluate a health recommendation system, over and above the information system that is used to generate the recommendations. Consumers prefer communication with their general practitioner or a hospital over communication with a fitness club and they prefer delivery through their general practitioner or via email over delivery by a fitness club. Accordingly, organizations planning to implement a health recommendation system must consider how and where to make this system accessible in terms of intermediaries.

Significant in this context and in line with previous research [64], we find that the general practitioner‘s advice plays an important role. Unlike other services, health-related information services rely on endorsements by the medical sector, and less medically oriented communication and delivery options, such as through a fitness center, are less appealing, possibly because non-medical parties may not be able to provide the necessary reassurance to consumers. This finding has considerable implications for the distribution and communication channels of health-oriented recommendation systems, which may be relatively hard to implement outside traditional health service channels. Specifically, companies could consider working together with doctors and / or hospitals as intermediaries and emphasize this collaboration in promoting such a health recommendation system. For example, the company Genetic Health, that offers personalized nutrition gene testing, requires consumers who wish to order a test kit to first talk to a genetic trained adviser. Also, Genetic Health offers consumers who ordered a test kit to investigate their personal genetic tolerance of medications (named Pharma Gene Test) to also have a full “ post test consultation” with one of their doctors. Similar options should possibly also be added in the case of personalized nutrition gene testing. It seems that since Genetic Health employs its own doctors, a possible extension could be to work with the consumers’ personal general practitioner in the system.

Furthermore, our results show that the intermediary context in which consumers first encounter a health recommendation system plays an important role in terms of which costs or benefits to emphasize and promote to consumers when introducing this service. The usefulness and value of employing a health recommendation system should be emphasized in an intermediary advice context, whereas its enjoyment potential is more relevant when the health recommendation system emerges in a context of participation out of one’s own interest

The information system domain itself also is vital. Foremost, organizations must make information available to consumers about which companies are involved in the process. Consumers dislike data handling if it is available to commercial food companies, do not favor designs by insurance companies, and disfavor production when it is specified in terms of branded food products. Somewhat more speculatively, these findings illustrate consumers’ reluctance to accept commercial applications of health recommendation systems.

More generally, complex information services, such as health recommendation systems, typically imply close one-to-one interactions with consumers that uniquely identify and address each consumer. To a great extent, such an intimate identification is key to optimizing and tailoring health recommendations, though it also may trigger greater consumer concerns about privacy risk. Thus, health recommendation systems confront a basic trade-off between usefulness and privacy risk: Greater privacy risk implies greater usefulness through more tailored recommendations. This trade-off poses a major challenge for the adoption of personalized health recommendation systems. We hope this study therefore also provides a further contribution toward developing new insights at the intersection of health sciences and information management, in particular, how best to assist consumers in adopting recommendation systems that allow them to develop healthier consumption patterns.

It is important to also mention some methodological limitations of this study. The first limitation relates to the health recommendation system options used in the study. Despite the fact that these were carefully selected based on discussions with experts, future research should still address if some other options are worth considering. This could possibly also be extended with expert and consumer focus group discussions. Second, a similar issue relates to omitted variables that might be incorporated in future studies. For instance, respondents’ technological awareness, health status, and possibly personality characteristics (e.g., curiosity, innovativeness) might be relevant to include. Third, we asked respondents to rate their intention to use the health recommendation system (the dependent variable) and not to actually make a choice. This could also be a possible future extension of this study. Lastly, the sample size of the current study is not very large. Although, we do find significant effects, future research could consider additional / alternative data collection methods to increase the sample size and allow for greater segmentation of respondents.

## Conclusions

We find support for our conceptual model. Specifically, we developed and tested a model of consumer’s intentions to use health recommendation systems. In an application to personalized nutritional advice, we find empirical support for the hypotheses that the different options in both the intermediary and information systems domains of a health recommendation system influence consumers’ intentions to use such as system and that these effects are mediated by consumers’ cost-benefit perceptions for effort, privacy risk, usefulness, and enjoyment. Furthermore, the participation advice of an intermediary that introduces consumers to the health recommendation system affects consumer’s orientation towards usefulness vs. enjoyment in determining their intention to use the recommendation system.

## Competing interests

SW, AR, BD and HvT declare that they have no competing interests.

## Authors’ contributions

SW and AR analyzed the data. SW and BD drafted the manuscript and contributed to the data collection. HvT provided expertise related the design of the study, interpretation of the results and assisted in writing the manuscript. AR assisted in writing the manuscript. All authors read and approved the final manuscript.

## Pre-publication history

The pre-publication history for this paper can be accessed here:

http://www.biomedcentral.com/1472-6963/13/126/prepub

## Supplementary Material

Additional file 1 Structure task of main study. Click here for file

## References

[B1] Liu DR Shih YY Integrating AHP and data mining for product recommendation based on customer lifetime value Inf Manage 2005 42 387 400 10.1016/j.im.2004.01.008

[B2] Xiao B Benbasat I Consumer decision support systems for e-commerce: design and adoption of product recommendation agents Manag Informat Syst Quarterly 2007 31 317-209

[B3] Brug J Oenema A Campbell M Past, present and future of computer-tailored nutrition education Am J Clin Nutr 2003 77 1028S 1034S 12663313 10.1093/ajcn/77.4.1028S

[B4] Kreuter MW Farell D Olevitch L Brennan LK Tailored health messages: Customizing communication with computer technology 1999 Mahwah, New Jersey: Lawrence Erlbaum Associates

[B5] Watzke JH German JB Moskowitz HR Personalizing Food An Integrated Approach to New Food Product Development 2009 New York, USA: Moskowitz Jacobs, Inc., White Plains 133 174 I. Sam Saguy, Hebrew University of Jerusalem, Rehovot, Israel; Tim Straus, The Turover Straus Group, Inc., Springfield, Missouri, USA CRC Press

[B6] Dijkstra A de Vries H Roijackers J Long-term effectiveness of computer-generated tailored feedback in smoking cessation Health Educ Res 1998 13 207 214 10.1093/her/13.2.207 10181019

[B7] Van Sluijs EMF van Poppel MNM Twisk JWR Chin A Paw MJ Calfas KJ van Mechelen W Effect of a tailored physical activity intervention delivered in general practice settings: Results of a randomized controlled trial Am J Public Health 2005 95 1825 1831 10.2105/AJPH.2004.044537 16186461 PMC1449443

[B8] Felfernig A Friedrich G Jannach D Zanker M An integrated environment for the development of knowledge-based recommender applications Int J Electron Commerce 2006 11 11 34 10.2753/JEC1086-4415110201

[B9] Joost HG Gibney MJ Cashman KD Personalised nutrition: status and perspectives Br J Nutr 2007 98 26 31 10.1017/S0007114507685195 17381877

[B10] Punj GN Moore R Smart versus knowledgeable online recommendation agents J Interact Mark 2007 21 46 60 10.1002/dir.20089

[B11] Crosby LA Evans KR Cowles D Relationship quality in services selling: an interpersonal influence perspective J Marketing 1990 54 68 81 10.2307/1251817

[B12] Ryan-Harshman M Vogel E Jones-Taggart H Nutritional genomics and dietetic professional practice Can J Diet Pract Res 2008 69 177 182 10.3148/69.4.2008.177 19063807

[B13] Dellaert BGC Dabholkar PA Increasing the attractiveness of mass-customization: the role of complementary online services and range of options Int J Electron Comm 2009 13 43 70 10.2753/JEC1086-4415130302

[B14] Prahalad CK Ramaswamy V Co-creation experiences: the next practice in value creation J Interact Mark 2004 18 5 14

[B15] Gollust SE Gordon ES Zayac C Griffin G Christman MF Pyeritz RE Wawak L Bernhardt BA Motivations and perceptions of early adopters of personalized genomics: perspectives from research participants Public Health Genomics 2012 15 22 30 10.1159/000327296 21654153 PMC3225236

[B16] Ricci F Werthner H Introduction to the special issue: recommender systems Int J Electron Comm 2006 11 5 7

[B17] Senecal S Nantel J The influence of online product recommendations on consumers’ online choices J Retailing 2004 80 159 169 10.1016/j.jretai.2004.04.001

[B18] Rousseau DM Psychological and implied contracts in organizations Employee Responsibilities and Rights Journal 1989 2 121 139 10.1007/BF01384942

[B19] Zeithaml VA Consumer perceptions of price, quality, and value: a means-end model and synthesis of evidence J Marketing 1988 52 2 22 10.2307/1251446

[B20] Tarrant C Stokes T Baker R Factors associated with patients’ trust in their general practitioner: a cross-sectional survey Br J Gen Pract 2003 53 798 800 14601357 PMC1314714

[B21] Babin BJ Darden WR Griffin M Work and/or fun: measuring hedonic and utilitarian shopping value J Consum Res 1994 20 644 656 10.1086/209376

[B22] Novak TP Hoffman DL Duhachek A The influence of goal-directed and experiential activities on online flow experiences J Consum Psychol 2003 13 3 16

[B23] Murthi BPS Sarkar S The role of the management sciences in research on personalization Manage Sci 2003 49 1344 1362 10.1287/mnsc.49.10.1344.17313

[B24] Vesanen J Raulas M Building bridges for personalization: a process model for marketing J Interact Mark 2006 20 5 20

[B25] Adomavicious G Tuzhilin A Personalization technologies: a process-oriented perspective Commun ACM 2005 48 83 90

[B26] Pavlou PA Liang H Xue Y Understanding and mitigating uncertainty in online exchange relationships: a principal-agent perspective MIS quarterly 2007 31 105 136

[B27] Culnan MJ Armstrong PK Information privacy concerns, procedural fairness, and impersonal trust: an empirical investigation Organ Sci 1999 10 104 115 10.1287/orsc.10.1.104

[B28] Awad NF Krishnan MS The personalization privacy paradox: an empirical evaluation of information transparency and the willingness to be profiled online for personalization MIS quarterly 2006 30 13 28

[B29] Robinson SL Trust and breach of the psychological contract Adm Sci Q 1996 41 574 599 10.2307/2393868

[B30] Dinev T Hart P An extended privacy calculus model for e-commerce transactions Inform Syst Res 2006 17 61 80 10.1287/isre.1060.0080

[B31] Davis FD Perceived usefulness, perceived use, and user acceptance of information technology MIS Quarterly 1989 13 319 340 10.2307/249008

[B32] Phelps J Nowak G Ferrell E Privacy concerns and consumer willingness to provide personal information J Public Policy Mark 2001 19 27 41

[B33] Rabino I Genetic testing and its implications: human genetics researchers grapple with ethical issues Science, Technology & Human Values 2003 28 365 402 10.1177/0162243903028003002 16208885

[B34] Koch M Möslein KM Identities management for e-commerce and collaboration applications Int J Electron Comm 2005 9 11 29

[B35] Bhattacherjee A Individual trust in online firms: scale development and initial test J Manage Inform Syst 2002 19 211 241

[B36] Davis FD Bagozzi RP Warshaw PR User acceptance of computer technology: a comparison of two theoretical models Manage Sci 1989 35 982 1003 10.1287/mnsc.35.8.982

[B37] Rogers EM Diffusion of Innovations 2003 5 New York: The Free Press

[B38] Childers TJ Carr CL Peck J Carson S Hedonic and utilitarian motivations for online retail shopping behavior J Retailing 2001 77 511 535 10.1016/S0022-4359(01)00056-2

[B39] Moon JW Kim YG Extending the TAM for a world-wide-web context Inf Manage 2001 38 217 230 10.1016/S0378-7206(00)00061-6

[B40] Kirmani A Campbell MC Goal seeker and persuasion sentry: how consumer targets respond to interpersonal marketing persuasion J Consum Res 2004 31 573 582 10.1086/425092

[B41] Dabholkar PA Bagozzi RP An attitudinal model of technology-based self-service: moderating effects of consumer traits and situational factors J Acad Market Sci 2002 30 184 201

[B42] Van der Heijden H Factors influencing the usage of websites: the case of a generic portal in the Netherlands Inf Manage 2003 40 541 549 10.1016/S0378-7206(02)00079-4

[B43] Algesheimer R Dholakia UM Herrmann A The social influence of brand community: evidence from European car clubs J Marketing 2005 69 19 34 10.1509/jmkg.69.3.19.66363

[B44] Srivastava RK Alpert ML Shocker AD A customer-oriented approach for determining market structures J Marketing 1984 48 32 45 10.2307/1251212

[B45] Wendel S Dellaert BGC Situation variation in consumers’ media channel consideration J Acad Market Sci 2005 33 575 884 10.1177/0092070305277447

[B46] Wendel S Dellaert BGC Situation-based shifts in consumer website benefit importance: the joint role of cognition and affect Inf Manage 2009 46 23 30 10.1016/j.im.2008.11.001

[B47] Deci EL Ryan RM Intrinsic motivation and self-determination in human behavior 1985 New York: Plenum

[B48] Pelletier LG Fortier MS Vallerand RJ Brière NM Associations among perceived autonomy support, forms of self-regulation, and persistence: a prospective study Motiv Emotion 2001 25 279 306 10.1023/A:1014805132406

[B49] Bloch PH Richins ML A theoretical model for the study of product importance perceptions J Marketing 1983 47 69 81 10.2307/1251198

[B50] Davis FD Bagozzi RP Warshaw PR Extrinsic and intrinsic motivation to use computers in the workplace J Appl Soc Psychol 1992 22 1111 1132 10.1111/j.1559-1816.1992.tb00945.x

[B51] Lee MKO Cheung CMK Chen Z Acceptance of Internet-based learning medium: the role of extrinsic and intrinsic motivation Inf Manage 2005 42 1095 1104 10.1016/j.im.2003.10.007

[B52] Shang RA Chen YC Shen L Extrinsic versus intrinsic motivations for consumers to shop on-line Inf Manage 2005 42 401 413 10.1016/j.im.2004.01.009

[B53] Yang X Li Y Tan CH Teo HH Students’ participation intention in an online discussion forum: why is computer-mediated interaction attractive? Inf Manage 2007 44 456 466 10.1016/j.im.2007.04.003

[B54] Mathwick C Malhotra N Rigdon E The effect of dynamic retail experiences on experiential perceptions of value: an internet and catalog comparison J Retailing 2002 78 51 62 10.1016/S0022-4359(01)00066-5

[B55] Hoy MG Switch drugs vis-à-vis Rx and OTC: policy, marketing, and research considerations J Public Policy Mark 1994 13 85 96

[B56] Ling DC Berndt ER Kyle MK Deregulating direct-to-consumer marketing of prescription drugs: effects on prescription and over-the-counter product sales J Law Econ 2002 45 691 723 10.1086/368004

[B57] Trussell J Stewart F Potts M Guest F Ellertson C Should oral contraceptives be available without prescription? Am J Public Health 1993 83 1094 1099 10.2105/AJPH.83.8.1094 8342715 PMC1695161

[B58] McGowan ML Fishman JR Lambrix MA Personal genomics and individual identities: motivations and moral imperatives of early users New Genet Soc 2010 29 261 290 10.1080/14636778.2010.507485 21076647 PMC2976061

[B59] Suh B Han I Effect of trust on customer acceptance of Internet banking Electron Commer R A 2002 1 247 263 10.1016/S1567-4223(02)00017-0

[B60] O’Cass A Fenech T Web retailing adoption: exploring the nature of Internet users web retailing behavior J Retailing and Consumer Services 2003 10 81 95 10.1016/S0969-6989(02)00004-8

[B61] Fornell C Larcker DF Evaluating structural equation models with unobservable variables and measurement error J Marketing Research 1981 18 39 50 10.2307/3151312

[B62] Taylor S Todd PA Understanding information technology usage: a test of competing models Inform Syst Res 1995 6 144 176 10.1287/isre.6.2.144

[B63] Baron RM Kenny DA The moderator-mediator variable distinction in social psychological research: conceptual, strategic, and statistical considerations J Pers Soc Psychol 1986 51 1173 1182 3806354 10.1037//0022-3514.51.6.1173

[B64] Henneman L Timmermans DRM van der Wal G Public experiences, knowledge and expectations about medical genetics and the use of genetic information Community Genet 2004 7 33 43 10.1159/000080302 15475669

