# Supplementary figures and images for: Consumers’ intention to use health recommendation systems to receive personalized nutrition advice
Source: BMC Health Serv Res. 2013 Apr 4;13:126. doi: 10.1186/1472-6963-13-126 (PMC3623628; doi:10.1186/1472-6963-13-126)

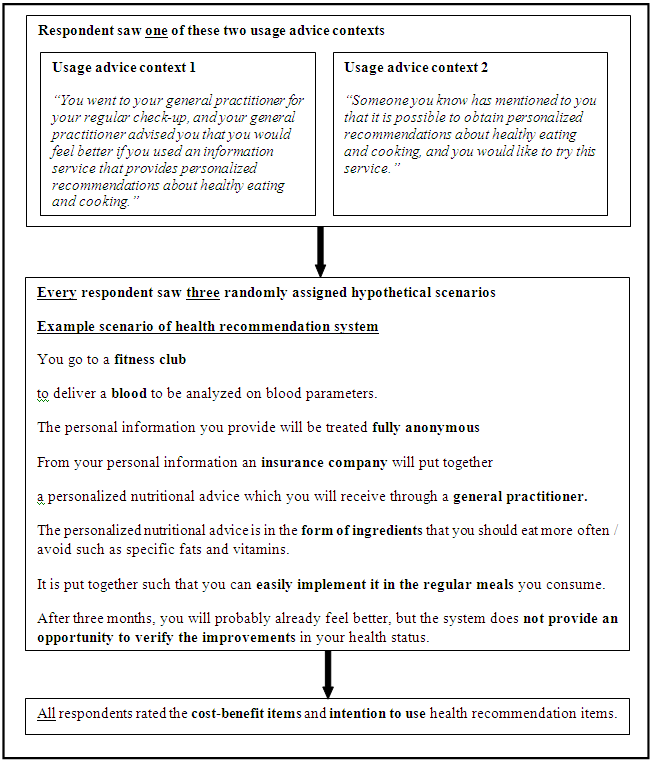

Supplement: Additional file 1 — Structure task of main study. [file 1472-6963-13-126-S1.doc]
